# Uncovering Hidden Predators: Thermal Drone Detection of Antarctic Fur Seals in Tussac Grass at South Georgia

**DOI:** 10.1002/ece3.73209

**Published:** 2026-03-09

**Authors:** J. Coleman, N. Fenney, P. N. Trathan, A. Fox, M. A. Collins, P. Hollyman

**Affiliations:** ^1^ British Antarctic Survey Cambridge UK; ^2^ Ocean and Earth Science National Oceanography Centre, University of Southampton Southampton UK; ^3^ School of Ocean Sciences Bangor University Anglesey UK

**Keywords:** Antarctic fur seal, thermal imagery, unmanned aerial vehicle, wildlife detection

## Abstract

Antarctic fur seals are an important predator in the Southern Ocean, with > 95% of the population breeding at South Georgia. Female seals generally pup on open beaches, but many move into long tussac grass to suckle offspring, where their presence can be concealed by vegetation. This makes it difficult to assess population changes, introducing considerable uncertainty. Broad‐scale, time‐efficient monitoring capable of detecting fur seals in tussac is therefore required to better understand population trends throughout the island, especially given recent reports of declines associated with reduced food availability, as well as important negative impacts from HPAI. This study utilises a fixed‐wing drone to provide both red/green/blue (RGB) imagery and thermal imagery for detecting fur seals in tussac grass as well as along beaches for assessing populations. Thermal sensors proved highly effective at detecting fur seals in tussac relative to RGB, with a much more efficient processing workflow. However, a combination of both is necessary to accurately identify seals across the range of coastal terrain in which they are found.

## Graphical abstract

We demonstrate that fixed‐wing drones equipped with dual thermal and RGB sensors can effectively detect Antarctic fur seals, including those concealed in tussac grass at South Georgia. Thermal imagery significantly improved detection rates compared with RGB alone, while a combined workflow provided the most accurate population estimates. This approach offers a scalable, time‐efficient method for monitoring a declining Southern Ocean predator and has broader applications for surveying cryptic wildlife in complex habitats.

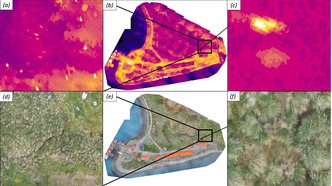

## 1 Introduction

**FIGURE 1:**
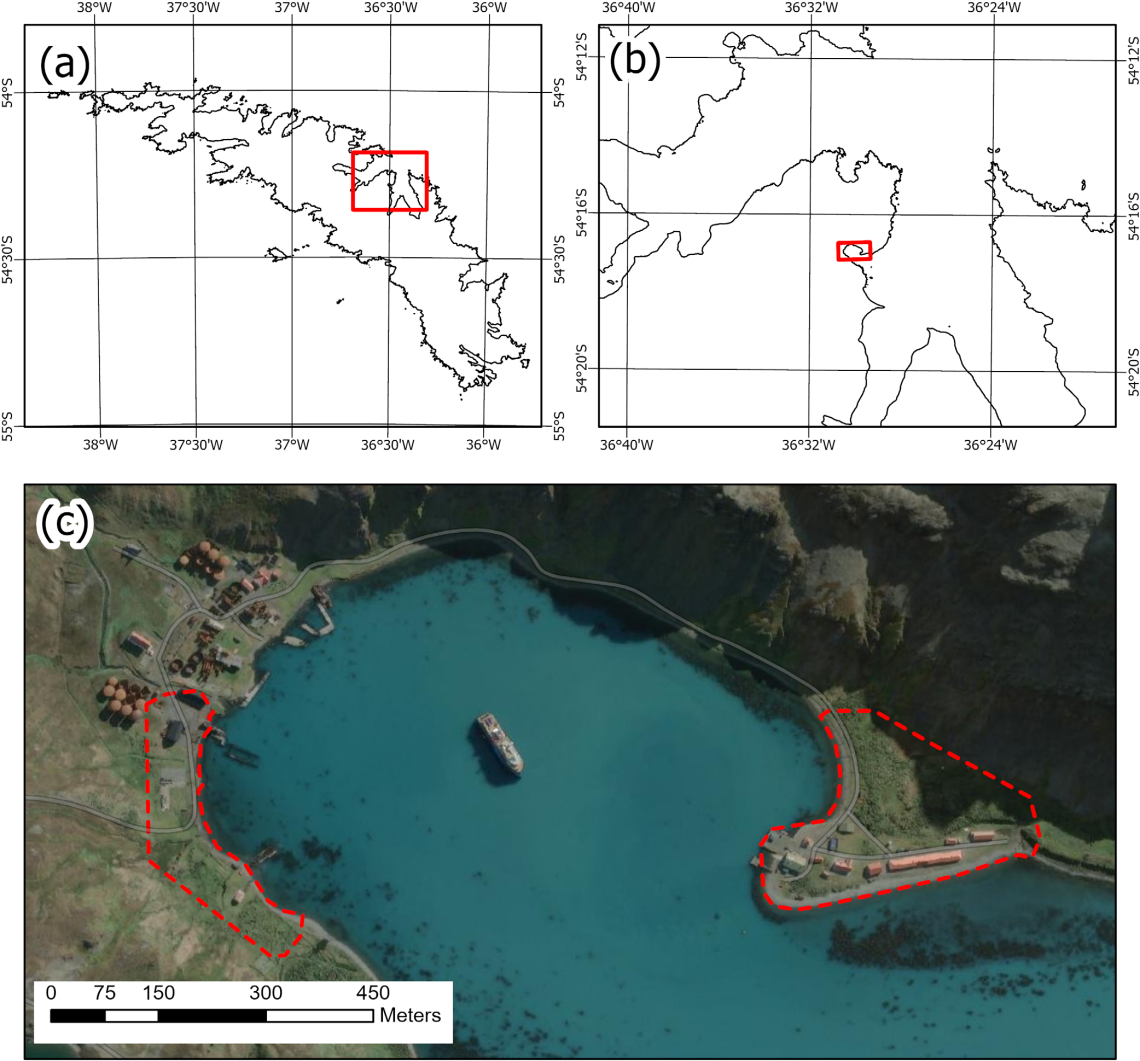
Map showing (a) South Georgia located within the Southern Ocean, (b) King Edward Cove situated within Cumberland Bay on the north coast of South Georgia and (c) Grytviken and King Edward Point survey sites with flight control site indicated (Microsoft product screen shot reprinted with permission from Microsoft Corporation Bing).

South Georgia is a Sub‐Antarctic island located in the Atlantic sector of the Southern Ocean (Figure 1). The surrounding waters are highly productive (Atkinson et al. 2001; Whitehouse et al. 2008) and provide habitat for a wide range of charismatic higher predator species comprising millions of individuals (Croxall et al. 1985; Coleman et al. 2024).

The waters surrounding South Georgia are experiencing a series of ecological changes associated with rapid warming (Meredith and King 2005; Stammerjohn et al. 2008; Whitehouse et al. 2008; Constable et al. 2014), coupled with the recovery of species that were over‐exploited historically (Basberg and Headland 2012; Zerbini et al. 2019; Calderan et al. 2020; Hollyman et al. 2021). One of these species, the Antarctic fur seal ( *Arctocephalus gazella* ; hereafter fur seal), was hunted to economic extinction (Payne 1977; Basberg and Headland 2012). Though numbers rapidly recovered close to pre‐sealing numbers (Staniland et al. 2011; Foley and Lynch 2020; Hoffman et al. 2022), parts of the population are now in decline (Forcada et al. 2023), possibly as a result of ecosystem change, density dependence, and/or competition with recovering populations of baleen whales (Trathan et al. 2021).

As important consumers of Antarctic krill (Reid and Arnould 1996; Barlow et al. 2002), Antarctic fur seal breeding output and diet are monitored at South Georgia to give insights into the status of the ecosystem and inform management decisions (Croxall and Prince 1987; Reid and Croxall 2001; Trathan et al. 2022; Coleman et al. n.d.). Although it is possible to correct for missing animals, monitoring of breeding pinnipeds should take place in a narrow window around peak pupping to give accurate data and reduce error of counts (McIntosh et al. 2018; Forcada et al. 2023). Population censuses of Antarctic fur seals are difficult due to the challenges of surveying post‐partum habitat which includes tall tussac grass. Whilst most fur seals are born on rocky beaches, many are born in territories in vegetation that can conceal adult seals and pups from observation (Gooday et al. 2018). As the season progresses and beaches fill, more seals move deeper into tussac (Nagel et al. 2021). Whilst a few individuals climb onto tussac mounds, many favour the shelter offered by the overarching tussac which forms tunnels where they are obscured from view. Whole island counts have been undertaken (Forcada et al. 2023) but have not specifically addressed the issue of fur seals in tussac being missed. Data from studies such as this could be used to correct future surveys. This issue is likely to be of increased importance since the 2007–2009 census (Forcada et al. 2023), as the eradication of introduced reindeer from South Georgia between 2011 and 2014 has enabled significant recovery of tussac cover in key coastal areas, expanding the extent of vegetated habitat available to fur seals (Heidbrink 2024).

Subantarctic islands are amongst the most wildlife‐rich areas on the planet, with large numbers of seabirds and seals utilising these predator‐free environments. These islands are remote, often with challenging terrain and dense vegetation, making monitoring of populations difficult and time‐consuming (Hegg et al. 2012). At South Georgia, routine fur seal monitoring is restricted to two locations, mostly in non‐tussac areas, each proximal to British Antarctic Survey research stations, with the last whole island census conducted in 2007–2009 (Forcada et al. 2023). More frequent, larger‐scale monitoring is necessary to better understand population trends of this species, especially given differing population trajectories in different parts of the island (Trathan et al. 2021) and the recent impacts of highly pathogenic avian influenza (HPAI) on the species (Banyard et al. 2024). Consequently, more efficient and accurate survey methodologies are required.

The use of remotely piloted aerial systems (RPAS, hereafter drones) for monitoring wildlife populations has increased globally (Vermeulen et al. 2013; Wich et al. 2016; Seymour et al. 2017) and as drones have become less constrained by weather and more affordable, this increase has also occurred in both Antarctica (Rümmler et al. 2016; Harris et al. 2019; Shah et al. 2020; Bello et al. 2022; Hinke et al. 2022) and South Georgia (Dickens et al. 2021; Coleman et al. 2024; Bamford et al. 2025). They offer a tool to improve the efficacy and efficiency of field studies globally (Hyun et al. 2020; Edney et al. 2023) including in otariid species (Allan et al. 2019; Hinke et al. 2022; Larsen et al. 2022, 2025).

Drone use has traditionally used RGB imagery (Allan et al. 2019; Larsen et al. 2022, 2025), though thermal sensors are now becoming available that facilitate improved detection of cryptic species in vegetation (Brunton et al. 2020; Hyun et al. 2020; Nazir and Kaleem 2021; Howell et al. 2022; Mirka et al. 2022; Virtue et al. 2023). Thermal sensors have been used to detect seabirds (Hinke et al. 2022; Virtue et al. 2023) and seals (Cronin et al. 2007; Seymour et al. 2017; Gooday et al. 2018; Hinke et al. 2022) in coastal habitats. However, their use has not always been more efficient than other traditional methods (Gooday et al. 2018; Hinke et al. 2022).

Autonomous, fixed‐wing drones, capable of being flown beyond visual line of site (BVLOS) allow significantly larger areas to be mapped rapidly from safe remote vantage points adjacent to colonies (Edney et al. 2023; Coleman et al. 2024). By equipping such drones with Forward Looking Infrared (FLIR) thermal sensors there is now the potential to maximise both the area mapped and the ability to detect fur seals within heavily vegetated areas where they may have previously been concealed from more traditional techniques (Gooday et al. 2018; Virtue et al. 2023).

In this study, for the first time, we use fixed‐wing drones to detect fur seals on South Georgia. Utilising the dual thermal/Red Green Blue (RGB) sensor of the AgEagle Ebee X drone we investigated whether thermal imagery could be used to count fur seals and increase accuracy of aerial counts, particularly within tussac. We also explored the relationship between flight height, image resolution, and area covered for both RGB and thermal sensors.

## 2 Methods

An AgEagle Ebee X fixed‐wing drone was used to fly two target areas, at Grytviken and King Edward Point (KEP) Research station, South Georgia on November 15, 2022 (Figure 1). The eBee X, a commercially available drone, was equipped with a AgEagle Duet T sensor. This combines an RGB, 20‐megapixel (MPx) S.O.D.A. (Sensor for Obstacle Detection and Avoidance) with a 640 × 512 Px thermal FLIR sensor for simultaneous thermal and RGB image collection.

When planning drone surveys, various factors need to be considered (Edney et al. 2023). This includes survey height and swathe width to increase data collection per unit effort, offsetting battery and flight duration. A further trade‐off is flight height and image resolution, which needs to consider any adverse disturbance effects on the target or other species (Edney et al. 2023). Finally, these factors all combine to determine image processing time and potentially the quality of the final results. For our survey, a key factor was the minimum pixel resolution at which a fur seal can be detected using the I/R sensor.

A single flight was carried out as close to dawn as possible to maximise contrast between seals and terrain in thermal imagery whilst also allowing for visible RGB data collection. The eBee X flight management software (FMS) eMotion was used for both planning and flight execution. The flight comprised a single survey at KEP, followed by four surveys of the same area at Grytviken. The KEP survey was flown once, with a thermal Ground Sample Distance (tGSD) of 10 cm, while the four repeat surveys at Grytviken were at four different GSD resolutions. These thermal GSD resolutions were 8, 10, 12 and 14 cm (Table 1). All surveys were carried out using an 80% / 60% forward/side overlap based on the thermal sensor to assess the most appropriate resolution for detection of seals using I/R.

**TABLE 1:** Specification of the surveys.

Survey	Target survey height above ground level (m)	Thermal sensor					RGB sensor		
		tGSD (cm)	Image width (m)	Image length (m)	No of images	Area covered (m^2^)	GSD (cm)	Image width (m)	Image length (m)
Grytviken 1	61.2	8	51	41	159	40,494	1.39	76	51
Grytviken 2	76.5	10	64	51	132	50,635	1.73	95	63
Grytviken 3	91.8	12	77	61	114	61,118	2.08	114	76
Grytviken 4	107.1	14	90	72	102	76,083	2.42	133	88
King Edward Point	76.5	10	64	51	272	84,878	1.73	95	63

Prior to any data collection flights, three test flights were carried out alongside an independent observer to monitor the impact of the drone on wildlife present in the area. No significant changes in behaviour were observed.

To maximise the quality of photogrammetric processing, a Trimble R9s global navigation satellite systems (GNSS) receiver was used and Precise point positioning (PPP) undertaken as in Coleman et al. (2024).

The photogrammetry and orthorectification workflows were undertaken in Pix4D (version 4.7.5). The photogrammetry workflow used the higher resolution RGB imagery to derive the position and orientation of both the RGB and thermal sensors during each image capture. For each survey tasking, this information was then used to derive a series of dense 3D point clouds of the surface from the RGB images and from which a triangulated mesh could be created. The triangulated meshes were then used to orthorectify both the RGB and thermal imagery, resulting in a series of geometrically corrected, georeferenced orthorectified mosaics.

Once the orthomosaics were produced, all visible seals were individually geotagged within ArcGIS Pro (Version 3.0.0). This was first done using RGB orthomosaics, then thermal orthomosaics, and then using both RGB and thermal to compare the visibility of seals. This combined count was assumed to be the truest value.

## 3 Results

All surveys were initiated from the Gull Lake plateau above Grytviken (Figure 1). Flying commenced at sunrise local time and lasted 47 min with the eBee X having travelled 36.2 km.

Antarctic fur seals, elephant seals ( *Mirounga leonina* ), and giant petrels (*Macronectes* spp.) were visible in both RGB and thermal imagery. Some man‐made structures displayed similar temperature properties to seals (Table 2), but the shape of these structures made them easy to remove. Both missed seals (false negatives) and misidentified objects/heat signatures (false positives) were apparent in both sets of imagery. The counting process of RGB images took longer than thermal imagery and resulted in a much lower detection rate of seals in the thick tussac grass. Moreover, manually counting fur seals in tussac using RGB imagery would be impractical when applied over medium to large areas.

**TABLE 2:** Results including false negative (FN) and false positive (FP) seals counted manually using only thermal imagery, RGB imagery, a combination of both, and automated counting.

	Thermal only			RGB			Combined full count
	Count	FN	FP	Count	FN	FP	
King Edward Point	158	2	10	142	8	0	150
King Edward Point Tussac^a^	28	0	0	20	8	0	28
Grytviken 8 cm	142	3	8	136	1	0	137
Grytviken 10 cm	160	4	15	149	1	1	149
Grytviken 12 cm	175	3	12	165	1	1	166
Grytviken 14 cm	188	1	10	180	2	1	181

^a^ Subcount of the King Edward Point count.

### 3.1 Thermal Based Manual Counts

In the thick tussac area behind King Edward Point research station the thermal sensor detected each of the seals present (28, see Table 2). Across both sites, errors from thermal imagery were most apparent at the beach. For example, several wet seals (returning from the sea) had less obvious thermal signatures and so were missed. Similarly, where female fur seals were nursing newborn pups, the two thermal footprints merged and just one animal was recorded. In contrast, thermal imagery also resulted in several false positives caused by seals moving from one resting location to another, resulting in an additional thermal footprint where the ground had been warmed.

### 3.2 RGB Manual Count

**FIGURE 2:**
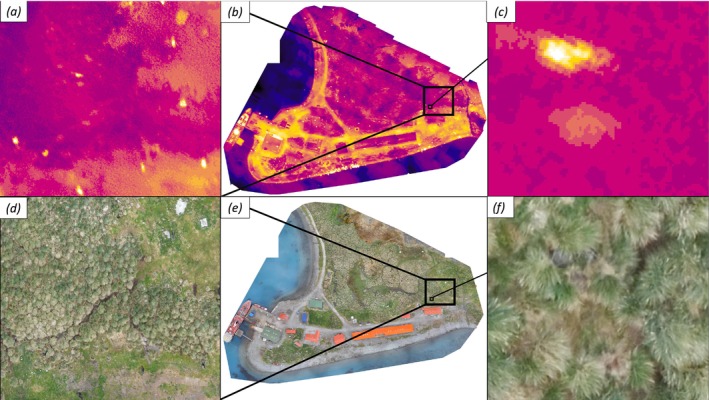
Thermal and RGB imagery of King Edward Point research station and surrounding tussac. Orthomosaics generated within Pix4D in thermal (b) and RGB (e) with close ups (a, c, d, f) demonstrating the contrast of identifying seals in tussac from different sensors.

Across both sites, at all GSDs, RBG count errors were associated with vegetated areas where seals lay beneath or partially hidden within tussac grass (Figure 2). At KEP, where tussac grass is denser, of higher stature, and more prevalent, more seals were missed than at Grytviken (Table 2). At King Edward Point we found that 28 fur seals were present within thicker tussac, of which 8 (28.6%) were not counted in RGB imagery but were visible with thermal.

### 3.3 Combined Count

Both false positives and false negatives were identified from thermal imagery when supplemented with RGB imagery. Similarly, seals missed within the tussac from RGB counts were easily recognised by thermal signature, and false positive targets (with no thermal signature) could be removed. The counting process of thermal followed by RGB was quicker than RGB alone.

### 3.4 Image Resolution

**FIGURE 3:**
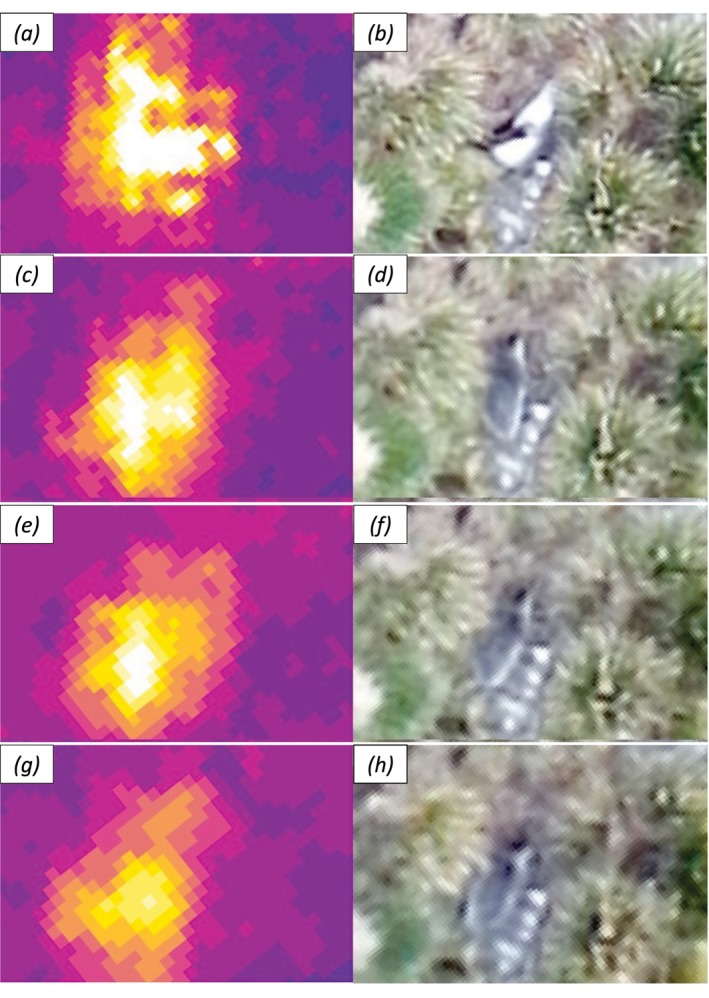
Thermal (left) and RGB (right) imagery with thermal GSD of 8 cm (a) and (b), 10 cm (c) and (d), 12 cm (e) and (f), and 14 cm (g) and (h).

Thermal image clarity was least at 8 cm tGSD (Figure 3), possibly because it was later in the day and therefore warmer, or as a result of the faster relative speed of the platform to the ground, due to the lower operating altitude. However, seals could still be identified, albeit with slightly larger footprints resulting from this apparent blurring. In comparison, RGB image clarity was least at 14 cm tGSD resolution and missed the most seals, although the 14 cm tGSD thermal images captured these targets. False positives were seen in RGB imagery at 10, 12, and 14 cm tGSD resolution at Grytviken.

## 4 Discussion

Drone‐based RGB surveys are highly effective for counting pinnipeds and other coastal wildlife in open, non‐vegetated habitats such as beaches and rocky shorelines, where detection rates are high and validation against ground counts is strong (Goebel et al. 2015; Hodgson et al. 2016; Krause and Hinke 2021). However, thermal imagery has the potential to reduce effort, increase detection rates, and reduce a known source of bias in population estimates in densely vegetated areas where wildlife are easily missed when only using RGB data or other traditional methods. There are still some errors associated with thermal imagery, and it should therefore not be used in isolation and should be used in parallel with RGB imagery. With more imagery, a semi‐automated workflow could be developed which counts the heat signatures associated with seals (Seymour et al. 2017).

We found manual RGB image analysis to be time consuming but effective on open ground; this was because identification of seals against a dark background was slow, requiring repeated changes in zoom factors. Previous studies have shown similar success identifying pinnipeds on open sparsely vegetated habitat (Hodgson et al. 2016; Krause and Hinke 2021; Hinke et al. 2022). On small open sites, this manual counting was impractical whereas with larger, well vegetated areas this would be unmanageable. More importantly, in heavily vegetated areas (e.g., KEP), RGB image analysis resulted in an undercounting of seals of 28.6%. With limited beach space available, much of their habitat comprises of tussac, especially as the season progresses (Nagel et al. 2021). Accurate counts within tussac habitat are vital for accurately estimating fur seal populations. Local context will therefore be important; for example, where fur seals numbers are particularly dense, movement into nearby tussac may be greater, with potential to miss larger numbers of seals. Whilst the analysis of RPAS imagery can be time consuming, the ability to cover large areas in a short amount of time is advantageous when compared to traditional ground counts when surveying otariid species, which have a short window of peak pupping (McIntosh et al. 2018; Forcada et al. 2023).

While our results demonstrate that a substantial proportion of fur seals occupying dense tussac habitat can be missed using RGB imagery alone (e.g., 28.6% at King Edward Point), this study was not designed to quantify how this bias scales across seasons or across South Georgia as a whole. Surveys were conducted early in the breeding season, approximately 1 month prior to peak pupping (Forcada et al. 2023) due to field time constraints, and during a poor season for fur seal numbers in Cumberland Bay (BAS unpublished data), resulting in lower than normal seal densities on beaches and reduced movement into tussac habitat. These factors, preclude robust population‐wide correction factors. Nevertheless, the consistent detection of concealed seals using thermal imagery identifies a clear source of error in current survey approaches and demonstrates the potential for thermal data to reduce this bias in future monitoring. This is not only true for Antarctic fur seals on South Georgia; errors, comparable to these are likely to exist whilst counting any species in dense vegetation such as tussac grass.

Errors associated with thermal imagery were biased towards beach areas where RGB imagery was most effective, especially in identifying cooler seals returning to land from the ocean. When multiple seals lay adjacent to, or on top of each other, for example nursing female fur seals with pup, a single, large heat signature was present, making undercounting a risk if using thermal only. False positives were associated with seal movements (Stander et al. 2021), for example in response to aggression, or avoidance of males, with multiple heat signatures present where seals had recently rested. False positives were easily checked against RGB imagery.

The use of the fixed‐wing drone in this study allowed for synchronous RGB and thermal data collection, accelerating the orthorectification process. The eBee X platform and high‐resolution sensors meant all data could be collected within one flight from one position, away from wildlife. The high‐resolution sensor allows greater GSD from higher altitudes and the horizontal propellers disperse sound perpendicular to the ground. This reduces disturbance to animals (Edney et al. 2023; Coleman et al. 2024) and allows for greater area coverage (Hyun et al. 2020; Heidbrink 2024). This is particularly important when working with thermal sensors, and well insulated animals when there is an optimal flying period shortly after dawn (Gooday et al. 2018).

The approach used here could be applicable to other target animal groups across heavily vegetated terrain (Hyun et al. 2020; Nazir and Kaleem 2021; Mirka et al. 2022). For example, the heat signature of giant petrels was clearly identifiable on the beach. This species breeds in loose colonies over large areas, with nests often camouflaged within the tussac. As a result, manually surveying nests can be time consuming. If thermal signatures of this species are also apparent within the tussac, then this method could increase counting efficiency and aid monitoring of this species over larger areas.

Despite a lower flight height, 8 cm tGSD thermal imagery was less clear than that flown at higher altitudes; this is possibly due to the rolling 30 Hz shutter of the thermal camera causing blurring when flying at higher speeds relative to the ground; this blurring was not apparent at 10 cm tGSD and above. As height and GSD increase, so does the area covered by the drone, maximising flight efficiency. However, at 14 cm tGSD, RGB resolution had decreased sufficiently that confirming thermal footprints was more difficult. The 14 cm survey achieved a swath width 76% greater than that of the 8 cm survey. Thus, when designing aerial surveys, understanding this trade‐off between GSD and area covered as well as image overlap is necessary for making informed decisions and maximising data collection. It is worth noting that fur seals range far from the beach (Nagel et al. 2021), significantly increasing the area required to be surveyed. As such, the monitoring methodology needs to be applicable at scale to support an operational monitoring programme.

Historically, population surveys for fur seals at South Georgia were undertaken from yachts or small boats (Boyd 1993) or aerial or vantage point RGB photographic surveys (Forcada et al. 2023). More recently, in other locations, drone‐based RGB surveys have replaced or complemented these approaches in open, non‐vegetated seal colonies, producing reliable population estimates (Hodgson et al. 2016; McIntosh et al. 2018; Larsen et al. 2022, 2025). These methods are time‐consuming and would rely on finding seals hidden within the tussac, which is not always possible. Where breeding seals are found amongst vegetation, for example across subantarctic islands, alternative, more accurate methods should be used, especially as vegetation recovers as a result of species eradication efforts (Springer 2018; Heidbrink 2024).

A combination of thermal and RGB image analyses will allow for better estimating fur seal populations, which is needed both at South Georgia (Trathan et al. 2021; Forcada et al. 2023) and at the South Shetland Islands (Krause et al. 2023). Understanding the relationship between numbers of fur seals on the beach and those in the tussac may also allow for correction factors to be applied to current small scale monitoring such as the photo counts on beaches at Maiviken (South Georgia), as part of BAS long‐term monitoring. If the relationship between numbers in the tussac and numbers on the beach is not linear between days/seasons then these photo counts may misinform decision makers about population trends. Whilst population declines have been reported at Bird Island (Forcada et al. 2023) it is not yet clear if this is representative across the whole of South Georgia or how significant impacts from the recent mortality events from HPAI (Banyard et al. 2024) have been on the population. Further investigation is therefore critical to understand the extent of these declines and the state of this species across the entire island.

## Author Contributions

**J. Coleman:** conceptualization (lead), data curation (lead), formal analysis (lead), investigation (lead), methodology (lead), writing – original draft (lead), writing – review and editing (lead). **N. Fenney:** conceptualization (lead), formal analysis (lead), funding acquisition (equal), investigation (lead), writing – original draft (equal), writing – review and editing (lead). **P. N. Trathan:** conceptualization (equal), funding acquisition (lead), project administration (equal), writing – review and editing (equal). **A. Fox:** conceptualization (equal), funding acquisition (lead), writing – review and editing (supporting). **M. A. Collins:** conceptualization (equal), funding acquisition (equal), project administration (supporting), supervision (supporting), writing – review and editing (supporting). **P. Hollyman:** conceptualization (equal), investigation (equal), project administration (lead), supervision (lead), writing – original draft (supporting), writing – review and editing (supporting).

## Funding

This work was supported by the Darwin Initiative (DPLUS109).

## Conflicts of Interest

The authors declare no conflicts of interest.

## Data Availability

Data is hosted at the Polar Data Centre https://doi.org/10.5285/303d8002‐712c‐4979‐b31b‐54e421100def.
